# Ultra-Processed Foods Controversy: Myths, Realities, and Policy Implications

**DOI:** 10.3390/nu18132118

**Published:** 2026-06-29

**Authors:** Antonis Zampelas

**Affiliations:** 1 Laboratory of Dietetics and Quality of Life, Department of Food Science and Human Nutrition, Agricultural University of Athens, 11855 Athens, Greece; azampelas@aua.gr; 2 Department of Life Sciences, School of Life and Health Sciences, University of Nicosia, Nicosia CY 2417, Cyprus

**Keywords:** ultra-processed foods, chronic diseases, nutritional policies, reformulation, additives

## Abstract

Ultra-processed foods (UPFs) have recently been associated with an increased risk of chronic diseases. However, this association remains controversial. The present commentary aims to clarify some of these controversies by summarizing the hypotheses linking UPFs to chronic diseases and critically examining the evidence regarding specific components of UPFs—such as saturated fat, added sugars, salt, and food additives—and their relationships with disease risk. Finally, the commentary discusses the implications of these findings for the development of dietary guidelines and public health policies at both global and national levels.

## 1. The Hypothesis

The role of food processing has emerged as a major focus of scientific inquiry, with accumulating evidence suggesting that the degree of processing may be associated with an increased risk of cardiovascular diseases, type 2 diabetes, and several types of cancer [1]. In particular, ultra-processed foods (UPFs) are manufactured by the food industry, which uses several additives, little or no whole foods, and a series of processes to increase their shelf life and make them extremely palatable. They are typically ready-to-eat or ready-to-heat products that require little or no culinary preparation and, consequently, are widely accessible and convenient [2,3]. UPFs are considered to be low quality, energy dense, high in refined starch, added sugar, saturated fat, trans fat, and sodium, and low in fiber, protein, vitamins, and minerals [2]. Therefore, the extent of food processing is increasingly recognized as an important dimension of diet quality, capturing qualitative aspects of foods that are not adequately reflected in traditional nutrient-based approaches [4].

Three hypotheses were generated recently by Monteiro and his colleagues concerning dietary patterns characterized by a high consumption of UPFs [5]. The first hypothesis is based on the observation that such patterns are progressively displacing traditional diets centered on whole foods and their culinary preparations. The second hypothesis is based on the observation that a diet high in UPFs results in the deterioration of diet quality, particularly in relation to chronic disease prevention. Proposed mechanisms that may promote overconsumption include high energy density, hyper-palatability, soft texture, and disrupted food matrices. In addition, such dietary patterns may reduce the intake of health-protective phytochemicals while increasing exposure to toxic compounds, endocrine disruptors, and potentially harmful classes and mixtures of food additives. The third hypothesis is based on the observation that a high UPF dietary pattern may increase the risk of multiple diet-related chronic diseases through a variety of interrelated mechanisms.

## 2. The Controversy

### 2.1. The NOVA Classification

The most widely used system in the literature to classify UPF is the so-called NOVA classification. NOVA classifies foods according to the nature, extent, and purpose of the industrial processing they undergo. It was designed by the Center for Epidemiological Studies in Health and Nutrition, School of Public Health, University of Sao Paulo, Brazil [6]. Food processing is defined as the set of physical, biological, and chemical processes to which foods are subjected after being separated from nature and prior to consumption or incorporation into dishes and meals. NOVA classifies all foods and food products into four groups. Group 1. Unprocessed or minimally processed foods. Group 2. Processed culinary ingredients. Group 3. Processed foods. Group 4 includes the so-called UPFs (Box 1). Ultra-processed foods are industrial formulations made entirely from or mostly from substances extracted from foods (oils, fats, sugars, starches, and proteins), derived from food constituents (hydrogenated fats and modified starches), or synthesized in laboratories from food substrates or other organic sources (flavor enhancers, colors, and several food additives used to make the product hyper-palatable). Manufacturing techniques include extrusion, molding, and preprocessing by frying.

Box 1 **Foods classified in the NOVA 4 Group.**

Fatty, sweet, savory or salty packaged snacks; cookies (biscuits); ice creams and frozen desserts; chocolates, candies, and confectionery in general; cola, soda and other carbonated soft drinks; ‘energy’ and sports drinks; canned, packaged, dehydrated (powdered), and other ‘instant’ soups, noodles, sauces, desserts, drink mixes, and seasonings; sweetened and flavored yogurts, including fruit yogurts; dairy drinks, including chocolate milk; sweetened juices; margarines and spreads; pre-prepared (packaged) meat, fish, and vegetables; pre-prepared pizza and pasta dishes; fast foods, from restaurants or boxed (e.g., burgers, hot dogs, and sausages); pre-prepared poultry and fish ‘nuggets’ and ‘sticks’; other animal products made from remnants; packaged breads and hamburger and hot dog buns; baked products made with ingredients such as hydrogenated vegetable fat, whey, emulsifiers, and other additives; breakfast cereals and bars; infant formulas and drinks and meal replacement shakes (e.g., SlimFast^®^); pastries, cakes, and cake mixes; and distilled alcoholic beverages (e.g., whisky, gin, rum, vodka, etc.)

### 2.2. Observational and Metabolic Studies

The vast majority of studies investigating the association between UPF consumption and the risk of non-communicable diseases (NCDs) use the NOVA 4 classification, and they are of a prospective cohort design. Most of these studies have linked overconsumption of UPFs to an increased risk of chronic diseases and their major risk factors, including dyslipidemia, hypertension, insulin resistance, and obesity. Some of the results of these studies have also suggested that the associations observed are not solely due to increased fat, carbohydrate, and protein intakes, but to the presence of several additives. It is important to note at this point that people who consume large amounts of UPFs may also exercise less, smoke more, have a lower income, poorer access to healthcare, and consume fewer fruits and vegetables, and sophisticated statistical adjustments cannot guarantee that all confounding factors have been removed. 

Evidence supporting the observations from prospective studies was provided by a metabolic study from Hall et al. [7]. In particular, this study investigated whether UPFs affect energy intake in 20 weight-stable adults. Subjects were randomized to receive either ultra-processed or unprocessed diets for 2 weeks, immediately followed by an alternate diet for 2 weeks. Meals were designed to be matched for present calories, energy density, macronutrients, sugar, sodium, and fiber. The results of this study showed that participants consuming, ad libitum, the diet composed predominantly of UPFs, consumed approximately 500 kcal/day more compared to an isocaloric diet of unprocessed foods, leading to weight gain. This finding suggested that characteristics of UPFs, including their high palatability, energy density, and texture, may facilitate passive overconsumption. Consequently, Hall et al. raised also the important question, as to whether the observed effect of UPF overconsumption on adverse health outcomes is driven primarily by their macronutrient composition (i.e., high content of fat and carbohydrates) or by other constituents such as additives and processing-induced modifications that enhance palatability and promote increased intake.

### 2.3. The Role of Additives

It is well established that certain additives can contribute to increased palatability, but an important clarification is warranted. Additives rarely act on their own as primary drivers of taste appeal. On the contrary, they form part of a broader technological design that enhances the overall sensory properties of food products. A range of additive classes may contribute to palatability, and these are not exclusively present in UPFs, but they are also present across a wide spectrum of industrially manufactured foods. These include: (i) flavor enhancers, most notably monosodium glutamate, which amplify umami perception and intensify flavor without necessarily increasing their macronutrient content; (ii) sweeteners (both caloric and non-caloric), which reinforce the preference for a sweet taste and may contribute to sustained or enhanced consumption irrespective of their energy content; (iii) flavorings, which reproduce or enhance complex organoleptic profiles (e.g., “cheese flavor,” “smoky,” and “bacon”) that would otherwise require more complex or costly raw ingredients; (iv) emulsifiers and stabilizers, such as lecithin or carboxymethylcellulose, which do not directly impart flavor but improve texture (e.g., creaminess, homogeneity, and “melt-in-the-mouth” sensations), thereby contributing to palatability; and (v) texture and crispness enhancers, which influence mechanical properties (such as crunchiness and mouthfeel) that are strongly associated with a hedonic eating experience.

The key point, regarding the effects of additives on the risk of NCDs,, is that the palatability of UPFs arises not only from their presence but also from the overall food matrix. In this context, macronutrient composition, including the relative proportions of carbohydrates and fat, can also enhance palatability and taste, irrespectively of the presence of additives. Currently, scientific understanding does not support the attribution of an independent “addictive” role to food additives on the basis of robust causal evidence. On the contrary, it does recognize that they are key tools in the industrial formulation of foods with high sensory appeal, which in turn may facilitate overconsumption. It is also important to acknowledge that the traditional dietary pattern of Southern Europe—commonly known as the Mediterranean diet—is extremely tasteful and palatable, yet adherence to this dietary pattern is associated with a reduced risk of NCDs. To add to the additives controversy, findings from the NutriNet-Santé study suggested that exposure to preservative food additives, which are widely used in industrial foods, is associated with a higher incidence of hypertension and cardiovascular diseases (CVD), independent of NOVA classification [8]. The same study also reported an association between exposure to synthetic food coloring additives and the incidence of type 2 diabetes [9]. NutriNet-Santé is a population-based prospective e-cohort study launched in 2009, which included 112,395 French-speaking participants aged ≥15 years with internet access. Dietary intake was assessed using three non-consecutive 24 h dietary records collected at baseline and every six months thereafter. Limitations include overrepresentation of women, individuals with higher educational levels, and participants with healthier lifestyles compared with the general French population. Also, the proportion of participants who received nutritional advice from a healthcare professional was unknown.

Overall, the role of food additives remains a matter of ongoing scientific debate. While prospective cohort studies can identify associations, they do not establish causality. In this context, a recent review questioned the hypothesis that food additives directly induce “hyper-palatability” properties or “food addiction”, suggesting instead that overeating is more closely linked to energy density and food availability than to additives, per se [10].

From a metabolic perspective, David Ludwig recently published an article suggesting that the physiological responses to food intake (e.g., postprandial alterations in blood glucose levels) can trigger not only homeostatic but also hedonic eating independently of palatability [11]. He further suggested that many commonly reported binge foods, such as bread, potato chips, popcorn, and sugary cereals, although relatively bland, can induce sharp increases in blood glucose and insulin levels, but 4 h postprandially, glucose levels decrease rapidly, which would increase hunger. At this time point, functional Magnetic Resonance Imaging showed an activation of the nucleus accumbens, a key subcortical structure within the ventral striatum that functions as the brain’s pleasure and reward center [12]. Of particular interest are also the findings of a recent study investigating the nutritional characteristics of foods with addictive potential [13]. Specifically, foods identified as having a higher addictive potential tended to be higher in carbohydrates, glycemic load, energy density, and fat. This finding again raises the question as to whether the main contributors to the overconsumption of UPFs are carbohydrates and fat rather than additives.

### 2.4. Classification or Misclassification

NOVA 4 is a valuable but imperfect public health tool. Its major strength is that it highlights features of modern industrial food systems that traditional nutrient-based approaches often miss. However, its principal weaknesses are inconsistent classification, limited reproducibility, heterogeneous food grouping, uncertain causal mechanisms, and occasional conflict with nutrient-based assessments. For example, this category does not only include foods high in simple and added sugars, saturated fat, and sodium but also foods that are permitted by the European Union to carry nutrition and health claims based on scientific evaluations by the European Food Safety Authority (EFSA). Examples of foods with nutrition and health claims approved by EFSA include n-3 fatty acid-enriched eggs and spreads, margarines with plant sterols/stanols, vitamin D-fortified plant-based drinks, iron-fortified breakfast cereals, and oat and barley cereals with beta-glucans. All these would be classified as NOVA 4. In addition, a recent study suggested that NOVA criteria do not allow for the functionally robust classification of foods [14]. In this study, French food and nutrition specialists completed an online survey in which they assigned foods to NOVA groups. The results indicated low inter-rater agreement, even when detailed ingredient information was available. 

Consequently, the nutritional and technological features of NOVA 4 products bear several criticisms, namely, (i) processing and nutritional quality are not synonymous. Some NOVA 4 foods (e.g., fortified plant milks, infant formulas, clinical nutrition products, certain whole-grain breads) may have favorable nutritional profiles despite being classified as ultra-processed. (ii) The same processing technologies are used across all NOVA groups. Techniques such as drying, milling, freezing, extrusion, and blending are employed in foods classified as minimally processed, processed, and ultra-processed, making the boundary between groups less clear than NOVA implies. (iii) Classification often depends on formulation rather than processing intensity. Two foods with similar nutrient profiles may fall into different NOVA categories because one contains an emulsifier or flavoring while the other does not. Therefore, the mainstream academic debate today should not be whether ultra-processed foods deserve attention, but whether “ultra-processing” is an independent causal determinant of disease or primarily a marker for other harmful characteristics of modern diets.

All the above arguments raise the question of whether the NOVA classification of UPFs effectively condemns a broad range of foods by grouping them into a single category with potentially far-reaching implications for the food industry and food supply without explicitly addressing the role of key nutritional drivers of health, namely, added sugars, total and saturated fat, and sodium. Interestingly, results from the US National Health and Nutrition Examination Survey (NHANES) suggested that diet quality assessed using the Healthy Eating Index was more consistently associated with cardiometabolic health than UPF intake assessed using the NOVA 4 classification [15]. Finally, it is noteworthy that, in the UK, the Scientific Advisory Committee (SACN) on Nutrition stated that it remains unclear as to what extent observed associations between UPFs and adverse health outcomes are explained by established relationships between nutritional factors and health outcomes on which the SACN has undertaken risk assessments [16]. Therefore, the consideration still remains as to whether these foods are inherently unhealthy due to processing or because of their high content in energy, saturated fat, salt, and/or free sugars.

## 3. Guidelines and Policy Implications

### 3.1. Ultra-Processed Food Contribution to Energy Intake

On the 2nd of December 2025, the San Francisco City Attorney initiated legal proceedings on behalf of the people of California, alleging that several major food corporations intentionally promoted addictive “hyper-palatable” UPFs. Echoing earlier lawsuits against the tobacco industry, this lawsuit argued that these companies acted in concert to exploit human biological drives and behaviors, thereby encouraging greater consumption of UPFs to enhance their sales and profitability. In parallel with the recent controversial Dietary Guidelines for Americans 2025–2030 [17], this lawsuit reflects a broader shift in public health discourse on dietary patterns and food processing. This raises the question of whether such legal and policy approaches represent an effective strategy to improve public health outcomes or whether they may instead contribute to public concern and unintended consumer confusion. It is important to acknowledge that, in the USA, the contribution of UPFs to total energy intake is high, reaching 65% in youth and 55% in adults [18]. In agreement with US findings, data from UK National Diet and Nutrition Survey reported similar estimates of UPF consumption, ranging from 51% as energy in adults to 68% as energy in children [19]. In contrast, in Italy, UPFs accounted for 23% of total energy intake in 2018–2020 in adults [20] and, in France, for 31.1% [21]. Moreover, it was also reported that, in Europe, the energy contribution from UPFs varied markedly across the 22 European countries included, ranging from 14 to 44%, being the lowest in Italy and Romania, while the highest was observed in the UK and Sweden. Fine bakery products and soft drinks were most frequently ranked as the main contributors. Countries with a higher sugar intake also reported a higher energy share from UPFs. It was noted, though, that no associations with Body Mass Index were observed [22]. Consequently, in Europe, the contribution of UPFs to the total energy intake is much lower than in the US and UK.

In Greece, data from the Hellenic National Nutrition and Health Survey (HNNHS) indicate that, in children and adolescents, the average percentage of total daily energy provided by UPFs was 39.8% [23]. Four major food groups were found to contribute > 10% of the total UPF intake: ready-to-eat/heat dishes (36.2%), sweet grain products (21.4%), savory snacks (15.4%), and sweets (12.9%). These provided 86% of the total UPF intake, with no significant differences between children’s weight status. In addition, children in the highest UPF tertile had significantly higher intakes of energy, carbohydrates, added sugars, saturated fats, polyunsaturated fats, and cholesterol, but lower intakes of protein compared to those in the lowest tertile [24]. Unpublished data from HNNHS also suggest that the contribution of UPFs to total energy intake was even lower in adults than in children and adolescents. Similarly to findings in children, no association between UPF intake and obesity were observed in adults.

### 3.2. Food Reformulation

It is therefore evident that there is not only a controversy over what we define as UPFs, but also over the fact that each country has its own profile of UPF consumption, and consequently, the effects of UPFs on health indices could vary. Therefore, food reformulation should not only be targeting UPFs.

Regarding food reformulation, an important question that arises is whether efforts to decrease added sugars, saturated fat, and sodium (main contributors to NCD elevated risk) should focus primarily on their reduction per se, or rather through replacement strategies involving alternative nutrients and/or additives, while ensuring that reformulated products are healthier, safe, of high nutritional quality, and affordable. This issue is illustrated by one of our studies [25], which assessed the level of compliance of savory baked goods (SBGs) producers in Greece with the Regulation (EU) 2019/649 for trans fatty acid (TFA) reduction and evaluated the subsequent impact on TFA, i-TFA, and saturated fatty acid (SFA) intakes. We observed that most SBG producers complied with the TFA upper limit of 2 g of i-TFA/100 g of fat in 2021—11.4% of SBGs exceeded the limit in 2021 compared to 31.1% in 2015. However, an unexpected increase in SFA content/100 g of product was also observed. This increase in SFA content would lead to increased intake, resulting in over half of the SBG consumers reaching 15% of their daily total energy intake in SFAs compared to 13.7% prior to reformulation. Another example of proposed substitution comes from a recent position statement by Hypertension Australia and the National Hypertension Taskforce of Australia. In particular, it was suggested that, if patients add salt to their food, they should make a 1:1 switch from regular salt to potassium-enriched salt, with a composition of approximately 75% sodium chloride and 25% potassium chloride, unless they are at risk of hyperkalemia [26]. However, it is questionable as to whether this strategy will contribute to a significant decrease in hypertension prevalence or if it will complicate this issue by impeding adaptation to lower-salt taste exposure and sustain a preference for saltier foods. Another example of a problematic substitution strategy comes from the USA where, in the 1980s, coincident with the onset of the obesity epidemic, nutrition policy shifted its focus towards fat restriction. In the Surgeon General’s Report on Nutrition and Health (1988), reducing dietary fat intake was identified as the main priority for dietary change because, at that time, fat provided more than twice as many calories in the diet as either protein or carbohydrates. Subsequent reformulation efforts by the food industry involved replacing fat with refined starches and sugars. However, more recently, carbohydrates, and especially simple carbohydrates, have increasingly been identified as a primary dietary concern. In this context, if reformulation is to remain a public health priority, it is essential first to identify the foods to be reformulated and second to carefully consider the nature and the extent of any proposed substitutions.

Finally, the critical policy question is whether reformulation should be voluntary or mandatory. In Greece, for example, in 2016, the Hellenic Food Authority (EFET) signed a Memorandum of Understanding (MoU) with the Hellenic Federation of Bakers to reduce salt content in all types of bread (excluding specialty breads containing cheese, olives, and/or sundried tomatoes). This MoU set an upper 1.2% salt limit target for non-prepackaged artisanal bread (as consumed) on a voluntary basis. Additionally, this initiative was supported by targeted communication efforts to bakers, including an article in a bakers’ magazine, dedicated awareness-raising events, and personal letters to the President of the Hellenic Federation of Bakers, in each regional unit, highlighting the importance of gradual salt reduction in bread. Unfortunately, the MoU and the related voluntary awareness activities were ineffective as a strategy to reduce salt in bread [27]. In particular, the mean salt content in bread in 2024 was 1.41%, compared to 1.32% in 2012 when the voluntary initiative started. Notably, only 19.4% of samples in 2024 contained ≤ 1.2% salt compared to 31.8% in 2012.

This prompts the consideration of key drivers beyond voluntary action. Several complementary measures may need to operate simultaneously and in a mutually reinforcing manner. These include the following: (i) Mandatory Regulations and Taxation: Taxation of soft drinks in the UK and mandatory TFA limits, served as key drivers of substantial, legally enforced product reformulation; (ii) Marketing and Promotion Restrictions: Regulations limiting the placement and advertisement of products high in fat, salt, and sugar could encourage manufacturers to reformulate in order to avoid sales restrictions and market disadvantages; (iii) Front of Pack Nutrition Labeling (FOPNL): Mandatory and highly visible interpretive FOPNL could create strong incentives for reformulation by discouraging negative consumer perceptions; and (iv) School Food Standards in Canteens and Dining Facilities: Nutritional procurement standards for schools frequently impose strict nutrient thresholds, prompting manufacturers to modify recipes across products supplied to the education sector.

## 4. Conclusions

Ultra-processed foods are not villains to be solely targeted, since, firstly, their heterogeneity, as classified by the NOVA classification is high, including foods promoting health indices; and secondly, their contribution to energy intake, and consequently nutrient and non-nutrient intakes, varies among different countries. Policies towards reformulation, either voluntary or, in some cases, mandatory, cannot stand alone. They should be designed and implemented in parallel with other policies, including FOPNL, promotion or restriction of food advertising, implementing food standards at school canteens, inclusion of nutrition modules in primary and secondary schools, according to the Food and Agriculture Organization guidelines, and taxation if necessary.

## Data Availability

No new data were created or analyzed in this study.
